# Pediatric Asthma: A Global Epidemic

**DOI:** 10.5334/aogh.2416

**Published:** 2019-01-22

**Authors:** Denise Serebrisky, Andrew Wiznia

**Affiliations:** 1 Department of Pediatrics, Jacobi Medical Center, Bronx, NY, US

## Abstract

The global prevalence, morbidity and mortality related to childhood asthma among children has increased significantly over the last 40 years. Although asthma is recognized as the most common chronic disease in children, issues of underdiagnosis and undertreatment persist. There are substantial global variations in the prevalence of asthma symptoms in children, with up to 13-fold differences between countries. The rising number of hospital admissions for asthma may reflect an increase in asthma severity, poor disease management and/or the effect of poverty. The financial burden of asthma is relatively high within developed countries (those for which data is available) spending 1 to 2% of their healthcare budget on this condition. Established in 1989, the Global Initiative for Asthma (GINA) attempts to raise awareness about the increasing prevalence of asthma, improve management and reduce the burden of asthma worldwide. Despite global efforts, GINA has not achieved its goal, even among developed nations. There are multiple barriers to reducing the global burden of asthma, including limited access to care and/or medications, and lack of prioritization as a public healthcare priority. In addition, the diversity of healthcare systems worldwide and large differences in access to care require that asthma management guidelines be tailored to local needs.

## Introduction

Pediatric asthma is a serious public health problem around the world. The World Health Organization estimated that approximately 300 million people currently have asthma worldwide, and with current trends rising, it is expected to reach 400 million by 2025 [1]. Nearly 250,000 people die prematurely each year from asthma, and most of all these deaths are preventable. Globally, death rates from asthma in children range from 0 to 0.7 per 100,000 people [2]. Among children, asthma is the most common chronic disease, ranking among the top 20 conditions worldwide for disability-adjusted life years in children [3].

## Increasing Prevalence

The most accurate information regarding the prevalence of asthma in children around the world is available from the International Study of Asthma and Allergies in Childhood (ISAAC). Phase I of this study was completed in 1994–1995 and involved over 700,000 schoolchildren aged 6–7 and 13–14 years from 56 countries. The study revealed marked geographic variations in the prevalence of asthma. Countries with low prevalence of asthma (2–4%) were mostly in Asia, Northern Africa, Eastern Europe, and Eastern Mediterranean regions, whereas countries with high prevalence (29–32%) were located in South East Asia, North America and Latin America [4,5] Phase III of ISAAC was conducted during 2000–2003 and involved over 1,100,000 school children from 98 countries [5,6,7]. Phase III of the study also showed significant geographic variations in asthma prevalence. While English-speaking countries and countries in Latin America had the highest rates of asthma per capita, the disease appeared to be less often recognized yet more severe in Africa, the Indian subcontinent and the Eastern Mediterranean. Several factors may explain these observations. In low-income countries and amongst ethnic minorities in developed countries, there may be less awareness that wheezing may be a symptom of asthma, even among those with frequent episodes [8]. This hypothesis is supported by findings showing that undiagnosed asthma among current wheezers was more common in children from lower income countries. Second, asthma care may be poorer in developing countries, leading to underdiagnosis. However, recent data showed that, unfortunately, suboptimal asthma management is a global phenomenon [9,10]. Differences in environmental exposures to air pollutants as well as infective agents may also contribute to the greater severity observed in developing countries [6].

## Morbidity and Mortality

Current statistics show substantial levels of morbidity and mortality among children with asthma. For example, worldwide trends indicate an increasing number of hospitalizations for asthma among young children, which can be attributed to increased severity, poor disease management, and poverty [1,11,12].

A survey of households in 29 countries in North America, Europe and Asia identified individuals with asthma who were symptomatic in the last year or were taking asthma medications [9]. Over 10,000 children and adults were interviewed. A substantial negative impact of asthma on patients’ lives was documented with a high number of school and work days lost, restrictions on lifestyle and requirements for urgent care. Another survey conducted in Norway showed that less than half of children admitted to a hospital for asthma had taken an inhaled corticosteroid on a regular basis, and in Turkey, a similar survey showed that only one fifth of children diagnosed with asthma were on daily anti-inflammatory therapy before the admission [13]. Interestingly, in all the studies, there was a clear overdependence on short-acting bronchodilators to manage acute asthma exacerbations. There are many reasons for these high rates of undertreatment with controller medications, including failure of medical providers to correctly classify the severity of their patient’s asthma, poor access to medications, and low rates of long-term adherence.

Childhood asthma accounts for many lost school days and may deprive children of both academic achievement and social interaction, particularly in underserved populations [14] and minorities [15]. The Asthma Insights and Reality (AIR) surveys, conducted worldwide from 1998 to 2001, were aimed at assessing variations in asthma severity and control and the state of asthma management with respect to the Global Initiative for Asthma (GINA) recommendations [9]. These surveys provided direct evidence of suboptimal asthma control in many patients worldwide, despite the availability of effective therapies, with long-term management commonly falling short of the goals of the GINA guidelines. For example, among patients with severe persistent asthma, use of anti-inflammatory therapy ranged from 26% in Western Europe to 9% in Japan; the percentage of asthmatic patients with at least one unscheduled emergency visit in the past year ranged from 47% in Japan to 29% in the United States [9].

Hospitalizations for asthma are an important measure of disease severity, but data from low and middle-income countries is mostly unavailable [16]. Countries that implemented asthma management plans have observed decreases in hospitalization rates [17,18] although they remain elevated among low socioeconomic status and minority populations [19]. Similarly, a recent European study showed that a large proportion of asthmatics had uncontrolled asthma; 57% of treated asthmatics were not well-controlled and 17% had an asthma-related hospitalization over the last year [20].

Disability-adjusted life years (DALYs), a metric that incorporates years of life lost as well as years lived with disability, is an accepted measure of disease burden [21]. The global ranking of asthma DALYs in children compared with other causes of DALYs is shown in Table 1. Unfortunately, asthma is among the top 20 causes of DALYs for children of all ages and among the top 10 causes in the mid-childhood (ages 5–14 years). In the older age group (10–19 years), asthma has become more common cause of DAYLs over the last decade.

**Table 1:** Global Ranking of Disability-adjusted Life Years in Children Due to Asthma Compared to Other Conditions.

Age Group	Year	

	1990	2010

1–4 years	18	18
5–9 years	6	8
10–14 years	6	3
15–19 years	16	12

It is estimated that asthma death rates have fallen worldwide by about one-third between 1990 and 2010; from 250 per million to 170 per million among males and from 130 per million to 90 per million among females [22]. However, there are large differences between countries. Data from the United States, Canada, New Zealand, Australia, Western Europe, Hong Kong and Japan show a mortality rate peak of 0.62/100,000 people in the mid-1980s among children and young adults with a progressive decline in the mid-2000s to mortality rates as low as 0.23/100,000 people [23,24]. These findings coincide with the introduction of national and international asthma management guidelines, suggesting the potential positive impact of policy measures to curtail asthma mortality [24].

## Pediatric Asthma and Air Pollution

Air pollution is particularly hazardous to the health of susceptible populations like children and the elderly. Children are at the highest risk because they inhale a higher volume of air per body weight than adults [25]. Numerous studies have shown that children living in environments near traffic have increased risk of asthma symptoms, asthma exacerbations, school absences, asthma hospitalizations as well as new-onset asthma [26,27,28,29]. These effects are larger in children living in metropolitan than those living in rural areas [30]. Recent data from the Aphekom project, a study focused on Improving Knowledge and Communication for Decision Making on Air Pollution and Health in Europe, indicated that near-road traffic-related pollution accounted for 15% of all pediatric asthma cases [31]. Rapid urbanization and industrialization throughout the world have increased air pollution and therefore population exposures. Worldwide, the main sources of outdoor pollutants are fuel combustion from vehicles, construction and agricultural operations, power plants and industries. Further complicating this problem, it is now recognized that global warming will increase the effect of outdoor air pollution on health [32,33].

Indoor levels of air pollutants (excluding environmental tobacco smoke) have also been related to asthma prevalence and/or symptoms [34]. Indoor environments depend on the quality of air that penetrates from outdoors and on the presence of indoor air pollution. Approximately half of the world’s population burns biomass for cooking and heating purposes, mostly in poorly ventilated areas [35]. The combustion process produces pollutants such as carbon monoxide, nitrogen oxide, sulfur dioxide and particular matters known to be risk factors for respiratory diseases such as asthma, obstructive lung disease and cancer [35,36]. The concentration of these pollutants is particularly hazardous since most children spend about 90% of their time in confined environments. The World Health Organization classifies indoor air pollution as the eighth most important risk factor for disease, responsible for approximately 3% of the global burden of asthma (up to 5% in low income countries). According to the Global Burden of Disease Study 2015, household air pollution from solid fuels accounted for about 5.5 million deaths worldwide in 2013 [37]. Many of these studies have demonstrated associations between exposure to indoor pollutants and the risk for several respiratory allergic conditions, including asthma.

Epidemiological studies have also shown an association between indoor dampness and mold with increased asthma incidence and prevalence regardless of the presence of atopy [38]. The respiratory health effects of smoke exposure have also been well documented. Studies have consistently shown that exposure to environmental tobacco smoke is an important risk factor for childhood asthma and as well as greater asthma morbidity in children of all ages [39,40,41,42].

## The Economic Burden of Pediatric Asthma

The monetary costs of asthma are substantial and include both direct medical costs (e.g., hospitalizations, emergency room visits, medical practitioner visits and medication), and indirect nonmedical costs (e.g., time lost from work or school, decreased productivity at work or school, premature death) [1,43]. Globally, the economic costs of asthma exceed those of tuberculosis and human immunodeficiency virus/acquire immune deficiency syndrome (HIV/AIDS) combined [44]. Developed economies spend 1 to 2% of their healthcare budget on asthma [1,45]. In the United States, asthma cost about $3,300 per patient each year in medical expenses. Medical costs associated with asthma increased from $48.6 billion in 2002 to $50.1 billion in 2007, and will likely keep growing. Unfortunately, about 40% of uninsured people and 11% of those with insurance cannot afford their asthma prescription medicines, leading to poorer outcomes [46]. Indirect costs are also substantial, in England, 69% of parents or partners of parents of asthmatic children reported having to take time off work because of their child’s asthma, and 13% had lost their jobs [47].

The economic burden of asthma disproportionately affects those with the most severe disease. In both Western and developing countries, patients with severe asthma are responsible for approximately 50% of all direct and indirect costs, even though they represent just 10 to 20% of all asthma sufferers [11,45]. By contrast, the 70% of asthma patients with “mild” disease account for only 20% of total asthma costs.

Despite the high cost of asthma care, several studies suggest that cost containing programs can be successfully implemented. A model of disease management conducted in Finland from 1994 2004 had a massive effect in reducing asthma mortality, morbidity and costs. The program focused on early diagnosis, active anti-inflammatory treatment, and networking between primary care providers and pharmacists. Health care costs were reduced from €500 million to €230 million [18]. Similarly, an education program implemented in the United States resulted in a 35% decrease in overall hospitalization rates, a 27% decrease in asthma-related visits to the emergency department, and a 19% decrease in outpatient visits; thus, suggesting a positive impact on care costs [48]. This type of program should be implemented in other countries while also adapting to local socio-economic conditions and cultural practices.

## Reducing the Global Burden of Pediatric Asthma

Unfortunately, there are many barriers to reducing the worldwide burden of asthma (Table 2). For the governments of much of the world population, asthma is not a healthcare priority. In developing countries, many patients have very limited access to care and essential medications. For example, in Brazil, the proportion of asthmatics using inhaled corticosteroids ranges only from 6–9% largely due to cost-related barriers [49]. In addition, asthma management must compete with other prevalent chronic illnesses for a share of available medical care resources. For example, in Africa, the most urgent healthcare priorities are poor nutrition, poor housing, and infectious diseases (especially HIV/AIDS). However, even in developed countries, access to care and ongoing management may be suboptimal, particularly for minority populations. Studies from the United States and Canada have found that minority children are less likely to be prescribed inhaled corticosteroids, even those with full prescription coverage [50,51]. Moreover, the increasing prevalence of asthma implies that as the number of asthma patients increases, asthma-related expenditures will become an even more important challenge.

**Table 2:** Barriers to Reducing the Burden of Asthma.


**Ecoominc** Poverty, inadequate resources, poor education, illiteracy, lack of sanitation and poor infrastructure.
**Health Care System** Low public health priority, poor health-care infrastructure, difficulty implementing guidelines developed in wealthier countries, limited availability of and access to medication, lack of patient education and resources.
**Cultural** Multiplicity of languages, religious and cultural beliefs, concerns about medications.
**Environmental** Tobacco, pollution, occupational exposures.


GINA guidelines stress that until there is a greater understanding of the factors that cause pediatric asthma and measures become available to reduce its prevalence, the focus should be on cost-effective management approaches that are available to most patients [24]. In addition to more research on the fundamental causes and pathogenesis of asthma, there are also urgent needs for: 1) effective patient management systems, particularly in primary care; 2) better and prompter diagnoses; 3) implementation of guidelines that are tailored to the local needs; 4) better referral and treatment patterns, including use of controller medications; and 5) cooperation between healthcare officials and primary care providers to develop, implement and sustain management programs that will work at a local level.

In summary, there has been a significant increase on the global prevalence, morbidity and mortality related to asthma among children over the last 40 years. Governments should commit to research, intervention and monitoring to reduce the burden of asthma in the world, develop cost-effective innovative strategies to prevent the disease and more integrated approach to treatment, thus avoiding premature and unwanted deaths and improving the quality of life of asthmatic children and their families. It is also important to continue the efforts of monitoring asthma prevalence and severity globally and to implement new actions to reduce the worldwide burden of asthma.
